# Sex-specific circRNA–miRNA–mRNA networks in peripheral blood mononuclear cells of patients with idiopathic pulmonary arterial hypertension: a pilot study

**DOI:** 10.3389/fgene.2025.1674894

**Published:** 2025-12-08

**Authors:** Yuan Li, Yuanyuan Sun, Yuxia Huang, Xiaoyi Hu, Ping Yuan

**Affiliations:** 1 Department of Pulmonary and Critical Care Medicine, the Second Xiangya Hospital, Central South University, Changsha, Hunan, China; 2 Department of Cardio-pulmonary Circulation, Shanghai Pulmonary Hospital, Tongji University School of Medicine, Shanghai, China; 3 Department of Pulmonary and Critical Care Medicine, Shandong Provincial Hospital Affiliated to Shandong First Medical University, Jinan, Shandong, China

**Keywords:** idiopathic pulmonary artery hypertension, sex difference, circRNA, miRNA, molecular signature

## Abstract

**Background:**

Idiopathic pulmonary artery hypertension (IPAH) is a life-threatening condition with obvious sex differences. Recently, the interactions among circRNAs, miRNAs, and mRNAs were found to be significant parts in IPAH. Thus, we want to explore whether RNA networks exist in IPAH via sex differential analyses.

**Methods and materials:**

Illumina HiSeq was used to detect differentially expressed (DE) RNAs in peripheral blood mononuclear cells (PBMCs) from six (three male) IPAH patients. Gene Ontology (GO) and Kyoto Encyclopedia of Genes and Genomes (KEGG) pathway analyses were employed for the exhibition of differential expression levels of circRNAs, miRNAs, and mRNAs. The competing endogenous RNA (ceRNA) networks among circRNAs, miRNAs, and mRNAs were constructed on the basis of the authoritative miRanda and TargetScan databases. Student’s t-test was employed, and *p* < 0.05 was applied to show significance.

**Results:**

There were 220 DE circRNAs (106 upregulated and 104 downregulated), 41 DE miRNAs (15 upregulated and 26 downregulated), and 160 mRNAs (65 upregulated and 95 downregulated) in the female group compared with the male group, respectively. Through interaction analyses among circRNAs, miRNAs, and mRNAs, upregulated has-miR-1304-3p might be sponged to hsa-circ-0005723 and hsa-circ-0030276 and downregulated hsa-miR-6859-3p was probably sponged to circ DGKD, to modulate 21 downregulation genes and six upregulation genes, respectively.

**Conclusion:**

There were sex differences in a comprehensive circRNA–miRNA–mRNA regulatory network in IPAH.

## Introduction

Idiopathic pulmonary artery hypertension (IPAH) is a tumor-like condition with high mortality and multiple pathogenic factors, which is caused by pulmonary vascular remodeling. It leads to an end stage of death due to right heart failure. The concrete mechanism is still worth exploring and demonstrating (Farber and Loscalzo, 2004; Forfia and Trow, 2013; Bienertova-Vasku et al., 2015; Courboulin et al., 2016; Miao et al., 2018; Wang et al., 2019; Zaiou, 2019).

The research has benefited from the rapid development of the tools, high-throughput techniques, and abundant RNAs from cells and tissues that could be observed to have vital roles in cellular activities (Hedlund and Deng, 2018; Hrdlickova et al., 2017; Kolodziejczyk et al., 2015; Papalexi and Satija, 2018; Stegle et al., 2015). Meanwhile, it is suggested that there are fewer than 2% of genome-encoding RNAs that are protein-coding RNAs (Consortium et al., 2007), while most of the total RNAs actually are non-coding RNAs (ncRNAs), containing circRNAs and miRNAs, which are divided based on size, structure, and shape (Hombach and Kretz, 2016). In addition, circRNAs and miRNAs are seen to be more novel than the traditional mRNAs to act as crucial parts in molecular aspect (Cai et al., 2019; Qiu et al., 2019; Xiong et al., 2019; Choi et al., 2015; Endo et al., 2013; Guo et al., 2012; Huang et al., 2014; Nie et al., 2019; Olivieri et al., 2016) and even modulate cell proliferation, migration, and energy metastasis, by regulating gene expression levels of pre-transcription, transcription, and post-transcription (Qiu et al., 2019; Chen et al., 2019; Chi et al., 2019; Peng et al., 2019; Xue et al., 2019). Meanwhile, more and more vesicles, secreted from bystander cells and containing many non-coding RNAs, exhibited essential physiological functions in pulmonary hypertension (Colombo et al., 2014; Thery et al., 2002; Aliotta et al., 2016; Gao et al., 2016; Letsiou and Bauer, 2018), such as the interactions between endothelial cells (ECs) and smooth muscle cells (SMCs) from pulmonary arterial hypertension (PAH) (Zhu et al., 2019). Thus, peripheral blood mononuclear cells (PBMCs) could also play a crucial role in the mechanism and development of IPAH, when ECs and SMCs were attached by PBMCs because of an immune reaction that was similarly demonstrated as an essential part of the progression of IPAH (Braam et al., 2016; Hashimoto-Kataoka et al., 2015; Takagi et al., 2018).

Therefore, we conducted transcriptome RNA sequencing (RNA-seq) in PBMCs from the three paired male and female IPAH patients. With the finding of sex differences in IPAH patients by our previous work (Yuan et al., 2017a; Yuan et al., 2017b), we aimed to comprehensively identify whether sex differences also existed in expressed circRNAs, miRNAs, and mRNAs of IPAH patients through the data of RNA-seq.

## Methods and materials

### Population

Six patients (three males) were enrolled in this study. All underwent right heart catheterization (RHC) to be confirmed as having IPAH at Shanghai Pulmonary Hospital from 1 August 2017 to 30 October 2017 by standard diagnosis as follows.

IPAH was diagnosed according to guidelines developed by the European Society of Cardiology and the European Respiratory Society for the diagnosis and treatment of pulmonary hypertension (PH) (Simonneau et al., 2019; Farber and Loscalzo, 2004). By using RHC data, the patients had been confirmed as having precapillary PAH, corresponding to a mean pulmonary arterial wedge pressure ≤15 mmHg and a mean pulmonary artery pressure ≥25 mmHg (Forfia and Trow, 2013). Second, exclusion of pulmonary hypertension due to drugs or any type of hormone treatment (Bienertova-Vasku et al., 2015). Third, radiological examination, including multi-detector computed tomography (CT) or conventional pulmonary cine-angiography, or magnetic resonance imaging, is crucial to exclude pulmonary hypertension as a result of left heart disease or lung disease or chronic thromboembolism (Courboulin et al., 2016). In addition, other forms of PAH must be excluded by immunology, serology, hematology, biochemistry, or ultrasound tests.

### Blood collection and human peripheral blood mononuclear cell isolation

From each participant, 4 mL of whole blood was collected into a freshly EDTA-coated tube with clinical practices and then diluted with isopyknic Dulbecco’s modified Eagle medium (with 10% fetal bovine serum) after transferring into another 15 mL centrifuge tube. PBMCs were isolated by Ficoll density gradient centrifugation. Briefly, diluted blood samples were added to the tube of 8 mL of Ficoll slowly and at a 45° angle and then centrifuged (2,000 rpm, 30 min, room temperature). After centrifugation, the second layer of cells was selected and transferred into another tube with 4 mL Dulbecco’s modified Eagle medium (with 10% fetal bovine serum). The mixture was washed twice with 4 mL of PBS and spun at 2,000 rpm, 10 min, room temperature each time. The supernatant was discarded, and 4 mL of Dulbecco’s modified Eagle medium (with 10% fetal bovine serum) was added.

### Total RNA sequencing

The data sequenced from Illumina HiSeq are called raw reads or raw data. The spline sequence in raw reads must be removed first, followed by the total RNA sequences of varying lengths. Then, quality control was performed on these sequences. N base sequences and sequences with a too-low Q20 ratio were removed, and data outside the range of 15–41 bp were removed from subsequent analysis. The resulting sequence is called clean reads. Clean reads were compared to the genome of the specified species, and the alignment rate was calculated. Total RNA sequencing reads included miRNA, circRNA, and mRNA. After obtaining clean reads, the first step is to use the software programs Bowtie and BLAST to compare the data of these RNAs with the circBase (Glazar et al., 2014), miRanda (John et al., 2004), and TargetScan (Agarw et al., 2015) databases and then carry out classification annotation of total RNAs. Differential screening of identified RNAs, as well as target gene prediction, gene function, and pathway enrichment, were performed in the standard process via Gene Ontology (GO, http://www.geneontology.org) and Kyoto Encyclopedia of Genes and Genomes (KEGG, https://www.genome.jp/kegg). For unannotated reads post-classification, *de novo* RNA prediction is performed using bioinformatics pipelines (Xie et al., 2021; Friedlander et al., 2008).

### Establishment of RNA interaction pairs and competing endogenous RNA networks

Based on the expression levels of circRNAs, miRNAs, and mRNAs, Pearson’s correlation coefficients and *p*-values were calculated for miRNA-targets. Negatively correlated pairs with a *p*-value < 0.05 and Pearson’s correlation coefficient >0.8 were selected for further analysis. These were the predicted pairs for circRNAs-miRNA and miRNAs-mRNAs.

We then used the shared pairs from the predicted binding sites and the predicted pair from the expression of mRNA, circRNA, and miRNA for further analysis. MiRNA–mRNA and miRNA–circRNA pairs were shared to predict competing endogenous RNA scores (ceRNA) according to the competing endogenous RNA prediction principle, using the following formula: ceRNA score = MRE for mRNA–miRNA/MRE for circRNA–miRNA. Shared circRNA-mRNA pairs were predicted from expression data using Pearson’s correlation coefficient, and based on the ceRNA score principle, circRNA–mRNA pairs predicted from the ceRNA score were considered true ceRNA pairs. A regulated ceRNA network (circRNA, mRNA, and miRNA interactions of interest) was mapped using Cytoscape (V. 3.6.0) software based on established co-expression data.

### Statistical analyses

A Student’s t-test was conducted to compare two RNA sequencing variables, with statistical significance defined as a fold change (FC) ≥ 2 or ≤0.5 and *p* < 0.05. SPSS, version 23 (Statistical Package for Social Science, Chicago, IL, United States) was used for the analyses of data. *p*-values under 0.05 were employed. Competing endogenous RNA (ceRNA) networks were conducted as an alluvial plot.

## Results

### Profiling of differentially expressed circRNAs, miRNAs, and mRNAs

**FIGURE 1:**
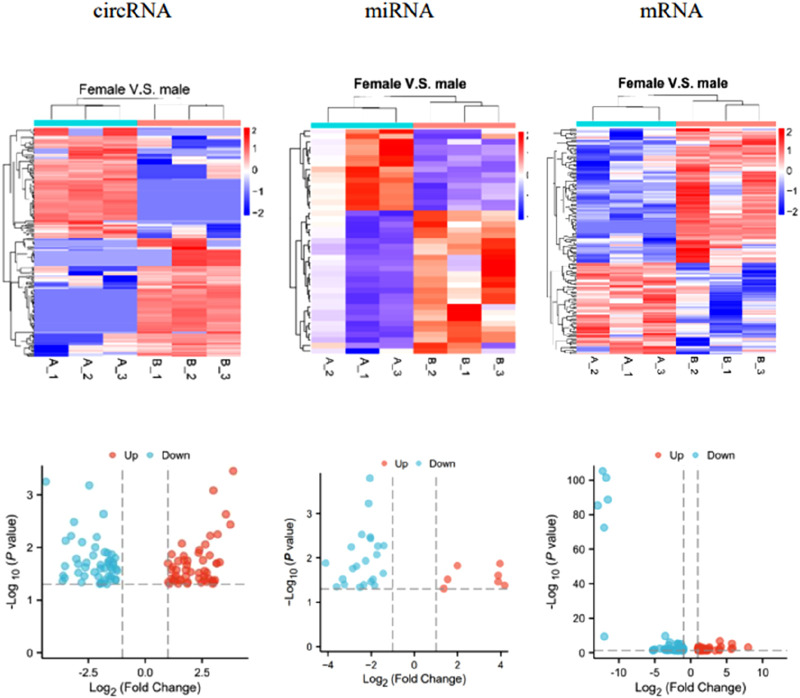
Heatmaps and volcano plots of circRNA, miRNA, and mRNA. |Fold change| > 2 and *p* < 0.05 were employed simultaneously.

Differential expression levels of circRNAs, miRNAs, and mRNAs are shown by a heatmap and a volcano plot (Figure 1). A total of 220 differentially expressed circRNAs were detected (106 upregulated and 104 downregulated), 41 differentially expressed miRNAs were detected (15 upregulated and 26 downregulated), and 160 differentially expressed mRNAs were detected (65 upregulated and 95 downregulated) in the female group compared with the male group, respectively. In terms of circRNA, miRNA, and mRNA, hsa_circ_0002908, hsa-miR-377-5p, and ADARB2 were the most upregulated, while hsa_circ_0009024, hsa-miR-6838-5p, and RPS4Y1 were the most downregulated, respectively.

### GO and KEGG pathway analyses in differentially expressed RNAs

Due to the large number of genes, only the top 30 terms were selected for the next analysis. GO and KEGG analyses were employed to more clearly and directly describe the enrichment of gene numbers in biological processes, cellular components, and molecular functions.

**FIGURE 2:**
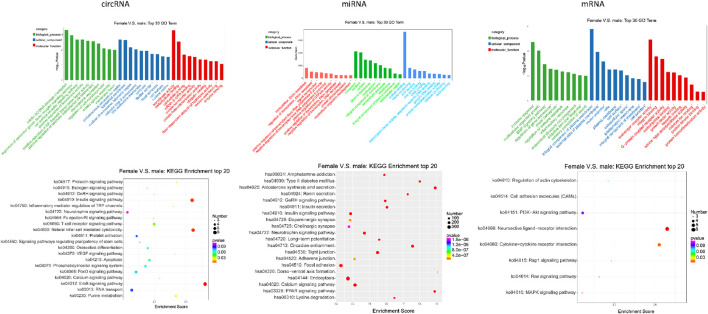
Top 30 enriched GO terms and top 20 enriched KEGG pathways of DE circRNA, DE miRNA, and DE mRNA.

As shown in Figure 2, in GO analysis, the differentially expressed circRNAs from female patients suggested that they had a main relationship to two biological processes (mitotic G2 DNA damage checkpoint and regulation of epidermal growth factor receptor signaling pathway), two cellular components (proteinaceous extracellular matrix and lysosomal membrane), and two molecular functions (transferase activity and carbohydrate binding), compared with those from male patients. Still in comparison to the results from the males, the upregulation of differentially expressed circRNAs from the females were principally related to two biological processes (protein sumoylation and nervous system development), two cellular components (focal adhesion and catalytic step 2 spliceosome), and one molecular function (transferase activity), while the downregulations were basically related to two biological processes (cell surface receptor signaling pathway and regulation of cell morphogenesis), one cellular component (centriole), and one molecular function (protein transporter activity).

As shown in Figure 2, GO analysis of the total miRNAs from the female group showed that the miRNAs were mainly related to one biological process (DNA-templated transcription), two cellular components (cytoplasm and nucleus), and one very significant molecular function (protein binding).

In addition, GO analysis also demonstrated that these differentially expressed mRNAs from the female group were mainly related to the biological processes of immune response, multicellular and organism growth, cellular components of integral components of the plasma membrane, and molecular function of scavenger receptor activity. Of those, the upregulations of differentially expressed mRNAs from the females were principally related to one biological process (sensory perception of sound), two cellular components (extracellular space and neuron projection), and one molecular function (growth factor activity), while the downregulations were related to five biological processes (immune response, inflammatory response, regulation of gene expression, regulation of immune response, and G-protein coupled receptor signaling pathway), one cellular component (integral component of plasma membrane), and one molecular function (G-protein coupled receptor activity).

KEGG pathway analysis revealed many important results. Figure 2 shows the top 20 pathways enriched in total differentially expressed circRNAs from the female group, but only half of them were significantly different from those of the male group. Of them, the insulin signaling pathway, natural killer cell-mediated cytotoxicity, and erbB signaling pathway were the most enriched pathways.

Similarly, KEGG pathway analysis revealed the top 20 pathways enriched in these miRNAs, all of which were of significance. Of them, the endocytosis was the first enriched pathway in the female group. Focal adhesion, calcium signaling pathway, and tight junction were the next most enriched.

In addition, the top 20 pathways enriched in differentially expressed mRNAs were selected in KEGG pathway analysis. Of them, there were two pathways showing significance in Figure 2: neuroactive ligand–receptor interaction and cytokine–cytokine receptor interaction.

### Competing endogenous RNA networks between miRNAs, circRNAs, and mRNAs

First, down–up regulation mapping was applied to the interactions between DE circRNAs and DE miRNAs via miRanda and TargetScan. As shown in Table 1, only two miRNAs, hsa-miR-1304-3p and hsa-miR-6859-3p, had relationships to DE circRNAs, of which the upregulated circRNA_18482 was associated with both miRNAs. Of those DE circRNAs, circRNA_32568 and circRNA_32158 originated from chromosome Y and chromosome X, respectively. Moreover, circRNA_08161 (hsa-circ-0005723) and circRNA_08414 (hsa-circ-0030276) had multiple target sites for has-miR-1304-3p. Thus, with the down-up logistic links and circRNA original chromosome, the upregulated hsa-miR-1304-3p associated with circRNA_08161 (has-circ-0005723) and circRNA-08414 (hsa-circ-0030276), and the downregulated has-miR-6859-3p associated with circDGKD was considered as a pre-sponge to modulate the pathologic development of IPAH.

**TABLE 1:** Down–up mapping of differentially expressed circRNAs and differentially expressed miRNAs.

CircRNA	Regulation	Mature name	Chromosome	Strand	CircRNA length	*p*-value	Target miRNA	Regulation	Total score^a^	Total energy^b^	Target sites
circRNA_32568	Down	NA	ChrY	+	22,911	5.8E−11	hsa-miR-1304-3p	up	175	−32.75	1
circRNA_09154	Down	NA	Chr14	−	2050	0.01590011			183	−36.02	1
circRNA_08161	Down	hsa-circ-0005723	Chr13	−	37,705	0.019163786			860	−160.02	5
circRNA_08414	Down	hsa-circ-0030276	Chr13	−	36,469	0.030389974			695	−127.39	4
circRNA_32158	Down	NA	ChrX	+	38,035	0.041811596			183	−34.61	1
circRNA_18482	Up	NA	Chr2	+	25,372	0.039025865	hsa-miR-6859-3p	down	177	−37.44	1

^a^ Total score: cumulative prediction score; bigger means more believable.

^b^ Total energy, cumulative complementary pairing matches free energy; smaller means more reliable; NA, not available.

**FIGURE 3:**
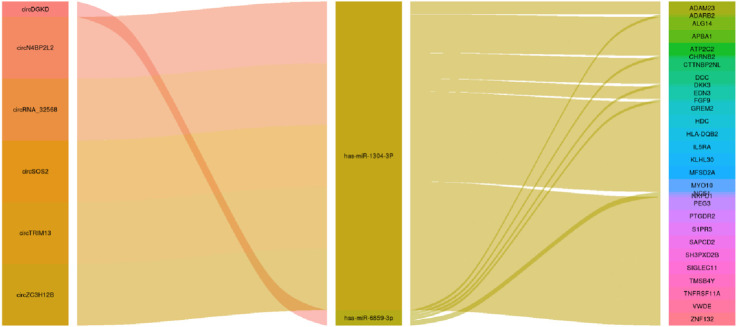
CeRNA networks of DE circRNAs, DE miRNAs, and DE mRNAs.

Next, the interactions between the two miRNAs (hsa-miR-1304-3p and hsa-miR-6859-3p) and DE mRNAs were evaluated through down-up regulation mapping. Twenty-four downregulated and six upregulated DE mRNAs were explored as the targets of has-miR-1304-3p and has-miR-6859-3p, respectively. Of those targets of has-miR-1304-3p, a literature review was employed to verify five confirmed DE mRNAs and five candidates associated with the pathologic development of IPAH (Table 2). Of those targets of has-miR-6859-3p, a literature review was employed to verify two confirmed DE mRNAs associated with the pathologic development of IPAH (Table 2). Finally, by merging the regulatory relationships between circRNAs (hsa-circ-0005723, hsa-circ-0030276, and circRNADGKD) and miRNAs (hsa-miR-1304-3p and hsa-miR-6859-3p), as well as miRNAs (hsa-miR-1304-3p and hsa-miR-6859-3p) and target mRNAs, a comprehensive circRNA–miRNA–mRNA regulatory network was built, as shown in Figure 3.

**TABLE 2:** Target differentially expressed mRNAs of has-miR-1304-3p and has-miR-6859-3p.

miRNA	Target mRNA	Regulation	Fold change^a^	*p*-value	Related to IPAH^b^	Note	References
hsa-miR-1304-3p (upregulated, 22 nt)							
	DCC	Down	Inf small	0.045087	Y	Secreted by smooth muscle cells and injured endothelial cells	Liu et al. (2017)
	GREM2	Down	0.242807	0.020425	Y	Participating in BMP4 signaling	Nolan and Thompson (2014)
	MYO10	Down	0.424778	0.001471	Y	Promoting the proliferation of smooth muscle cells	Zhang et al. (2020)
	PTGDR2	Down	0.445204	0.000712	Y	Related to attenuation of pulmonary hypertension	Chen et al. (2018)
	S1PR3	Down	0.321672	0.009555	Y	Involved in microvascular vasoconstriction	Meissner (2017)
	SH3PXD2B	Down	0.379297	0.001486	Y	Mediating endothelial motility	Mehes et al. (2019)
	MFSD2A	Down	0.460917	0.004738	Y	Involved in vasculature metabolism	Ungaro et al. (2017)
	PEG3	Down	Inf small	0.048681	Y	Regulating skeletal muscle growth	Dierick et al. (2016)
	SIGLEC11	Down	12.43713	0.006774	N	Preventing immune activation and involved in VEGF signaling	Karlstetter et al. (2017)
	TMSB4Y	Down	Inf small	1.12E-16	N	Regulating cell morphology and cell proliferation	Wong et al. (2015)
hsa-miR-6859-3p (downregulated, 23 nt)							
	DKK3	Up	2.752886	0.043492	Y	Modulating vascular smooth muscle cell differentiation	Karamariti et al. (2013), Wang et al. (2015)
	FGF9	Up	2.719154	0.024085	Y	Regulating PDGFRB signaling of smooth muscle cells	Dong et al. (2017)

^a^ Fold change, the female group compared to the male group; Inf small, infinitely small.

^b^ Y, related to participation in the IPAH mechanism.

^b^ N, not confirmed to participate in IPAH mechanism but could be a strong candidate; Ref, references.

## Discussion

In our study, we first reported the sex differences of circRNAs, miRNAs, and mRNAs expression in the PBMCs of IPAH patients. Compared to the male subgroup, the samples from female patients were more involved in the pathways related to IPAH mechanisms. Despite reported sex-specific miRNAs of PH, there was scant research regarding small RNAs of PBMCs from IPAH patients. Previous studies have concentrated on circulating miRNAs between PH patients and healthy controls.

Pulmonary vascular resistance (PVR) is the key factor related to PH. Kheyfets et al. (2017) identified 15 circulating miRNAs that correlated with the maximum change in PVRi between baseline and challenge conditions in a pediatric PAH population, of whom most were pediatric IPAH patients. Actually, microRNAs exhibited a sex-specific manner early in 2015. Wallace et al. (2015) demonstrated that miRNA-96 was a sex-specific participant in BMPR-II(R899X+/-) PASMCs from female mice and hPASMCs from female patients with PAH. Decreased miRNA-96 could target 5-HT1BR and promote serotonin-induced proliferation.

Muscle-related miRNAs were measured in circulating plasma after exercise. A study of plasma-based miRNAs in 10 male rowers measured muscle-related and dynamically regulated miRNAs following sustained aerobic activity, of which targeted mRNAs were involved in skeletal and cardiac muscle contractility (Baggish et al., 2011). Based on this study, Hicks et al. (2018) carried out another plasma profiling research of dynamically expressed miRNAs after exercise and demonstrated that hsa-miR-6859-3p existed in a sex-specific manner and was a kind of downstream regulator in the female sex, especially after exercise. As we all know, IPAH is a disease that severely damages the cardiopulmonary system, and cardiopulmonary exercise tests showed that women were more severely damaged with poor exercise endurance and low intensity (Yuan et al., 2017b). Therefore, we assumed that hsa-miR-6859-3p might affect the exercise system in IPAH patients, especially in females.

Sex is a known risk factor in IPAH, but the sex hormones present a paradox. Our study showed that immune-related pathways were more significantly enriched in the female patients than in the male patients. Actually, females generate more robust immunity than males (Wilkinson et al., 2022). It is worth noting that the chromosome XX mice were more likely to suffer certain autoimmune diseases than the chromosome XY mice in some studies (Sasidhar et al., 2012; Smith-Bouvie et al., 2008), which suggested a relationship between the chromosome X and autoimmunity. From single-cell transcriptome data, the patients with IPAH had a special neutrophil cluster characterized by higher MMP9 expression, which indicated the neutrophil-specific matrix metalloproteinases would participate in the pathogenesis process (Zhang et al., 2023). E2-ER signaling was considered an important axis involved in the pathological morphology of IPAH. In our study, the erbB signaling pathway showed that the top five pathways in the female group differed from those in the male group. Moreover, circZC3H12B, a downregulator in the female subgroup, was found to originate from chromosome X. The gene symbol of circZC3H12B was found in a new member of the ZC3H12 family (Wawro et al., 2019). ZC3H12B formed small, granule-like structures in the cytoplasm, which is an obvious characteristic of proteins involved in mRNA turnover. The overexpression of ZC3H12B inhibited proliferation by stalling the cell cycle in the G2 phase. This effect of ZC3H12B was also thought to be NYN/PIN dependent. CircRNAs perhaps had corrections with their originating genes (Li et al., 2018). Thus, circZC3H12B might affect ZC3H12B to participate in the morphology of pulmonary arterial cells. More interestingly, TMSB4Y, predicted as the downstream of hsa-miR-1304-3p, is confirmed from the Y chromosome. In male breast cancer, TMSB4Y was suggested as a candidate tumor suppressor (Wong et al., 2015). Overexpression of TMSB4Y promoted the aberrant cellular morphology and reduced cell proliferation. Further exploration discovered that it directly interacted with β-actin, the main component of the actin cytoskeleton and a cell cycle modulator. Taken together, our findings partly revealed similar results to previous studies and added more to the properties of sex differences in IPAH.

Huang et al. (2017) demonstrated that hsa-miR-1304-3p was decreased within the hippocampal tissues and increased in serum samples of temporal lobe epilepsy patients. Hsa-miR-1304-3p could also regulate ABCB1 transcriptional as well as translational levels. Luciferase assays verified that an miR-1304-3p/ABCB1 axis existed and might contribute to the mechanisms of drug resistance in temporal lobe epilepsy. Despite this literature, there have been few studies regarding its functional and pathological mechanical description. However, there were several studies about its mature sequence, hsa-miR-1304. Some reported that hsa-miR-1304 could participate in cell growth, migration, invasion, and apoptosis when sponged to some circRNAs in the LN229 cell line (Chen et al., 2020), the thyroid cancer cell (Zhang et al., 2019), and the papillary thyroid cancer cell (Pan et al., 2019). Some found that the number and viability of non-small cell lung cancer cells and colony formation were decreased, and cell apoptosis and G0/G1 phase cell cycle arrest were induced when overexpressed hsa-miR-1304 was developed via CRISPR/Cas9. Restoration of heme oxygenase-1 expression abolished the inhibition of miR-1304 on cell growth and rescued miR-1304-induced apoptosis in A549 cells. Thus, hsa-miR-1304 was regarded as a suppressor of heme oxygenase-1 expression, inhibiting cell growth and survival in non-small cell lung cancer cells (Li et al., 2017). Thus, we thought that hsa-miR-1304-3p in our findings might regulate cell proliferation, migration, and invasion as a suppressor.

SIGLEC11 (sialic acid-binding immunoglobulin-like lectin 11) is a cell surface signaling receptor to mediate anti-inflammatory and immunosuppressive signaling (Wang et al., 2010) and is always detected on macrophages such as Kupffer cells and microglia cells (Angata et al., 2002; Hayakawa et al., 2005). For example, Wang et al. (2010) demonstrated that human Siglec-11 expressed on murine microglia could reduce lipopolysaccharide (LPS)-induced gene transcription of pro-inflammatory mediators (interleukin-1beta, IL1, and nitric oxide synthase-2, NOS2) and impair phagocytosis via cross-linking stimulation and RT-PCR. Moreover, SIGLEC11 showed dependently inhibitory effects on VEGF expression of human macrophages (Karlstetter et al., 2017). Meanwhile, the VEGF signaling pathway is an important signal transduction pathway in IPAH, which tells us that SIGLEC11 could modulate the pathological mechanism of IPAH via the VEGF signaling pathway. Interestingly, its expression was also observed on fibroblasts, a type of mesenchyme-derived stromal cells, in both human and chimpanzee ovaries (Wang et al., 2011), indicating that SIGLEC11 could be a participant in the morphogen of the extracellular matrix (ECM). Increased ECM is a normal feature of IPAH, wherein it thickens the vascular media and facilitates cell adherence. SIGLEC11 might also regulate ECM in vessels to enhance the pathological features of IPAH. Thus, SIGLEC11 should be regarded as a strong candidate gene involved in IPAH, and the axis of (hsa-circ-0005723 and hsa-circ-0030276)/miR-1304-3p/SIGLEC11 is a promising RNA network in IPAH related to sex. However, the SIGLEC11 gene is not present in rodents, but SIGLEC16 exists, which shares a nearly identical N-terminal sequence to SIGLEC11. This point should be considered in the next verification (Cao et al., 2008).

## Limitations

The findings of this study must be seen in the light of some limitations. The first is the small size of the samples, based on which the results may have limited generalizability. The second concerns the lack of normal controls, basic experiment verification, and mechanism exploration, which we will address in future work.

## Conclusion

Compared with the male group, the expressed levels of circRNAs, miRNAs, and mRNAs were significantly different in the female group. The analyses of differentially expressed RNAs demonstrated that the RNA networks of hsa-circ-0005723 or hsa-circ-0030276/hsa-miR-1304-3p/mRNAs and circDGKD/hsa-miR-6859-3p/mRNAs might exist in IPAH related to sex.

## Data Availability

The datasets presented in this study can be found in online repositories. The RNA sequences were uploaded to the BioProject session of NCBI (The National Center for Biotechnology Information) with the primary accession codes 675373 (circRNAs and mRNAs) and 1176136 (miRNAs).
