# Supplementary material for: Sex-specific circRNA–miRNA–mRNA networks in peripheral blood mononuclear cells of patients with idiopathic pulmonary arterial hypertension: a pilot study
Source: Front Genet. 2025 Dec 8;16:1674894. doi: 10.3389/fgene.2025.1674894 (PMC12718719; doi:10.3389/fgene.2025.1674894)
Supplement: Supplementary file 1 [file Table1.docx]

| **Table S1 Baseline characteristics of patients with IPAH** | | | | |
| --- | --- | --- | --- | --- |
|  | Total  (n=6) | Male  (n=3) | Female  (n=3) | *P value |
| **General information** |  |  |  |  |
| Age/yds | 33.1 ± 13.8 | 42.5 ± 19.8 | 35.0 ± 15.0 | 0.233 |
| BMI/kg·m^-2^ | 22.6 ± 3.5 | 24.1 ± 2.9 | 21.1 ± 3.5 | 0.432 |
| 6MWD/m | 377.7 ± 174.1 | 398.1 ± 101.0 | 357.3 ± 174.1 | 0.488 |
| NT-proBNP/pg·mL^-1^ | 816 (138, 2197) | 1074 (467, 1967) | 558 (138, 2197) | 0.908 |
| WHO-FC, n (%) |  |  |  |  |
| I - Ⅱ | 2(33.3) | 1(33.3) | 1(33.3) |  |
| Ⅲ - IV | 4(66.7) | 2(66.7) | 2(66.7) |  |
| **Hemodynamics** |  |  |  |  |
| mPAP/mmHg | 63.9 ± 26.8 | 67.1 ± 26.1 | 60.7 ± 26.8 | 0.881 |
| mPAWP/mmHg | 7.0 ± 3.4 | 7.4 ± 2.6 | 6.6 ± 3.4 | 0.223 |
| mRAP/mmHg | 3.7 ± 2.2 | 4.5 ± 2.7 | 2.9 ± 2.2 | 0.263 |
| PVR/Wood units | 14.6 ± 5.6 | 12.6 ± 7.1 | 16.6 ± 5.6 | 0.963 |
| CI/L·min^-1^·m^-2^ | 2.5 ± 0.5 | 2.6 ± 0.7 | 2.4 ± 0.5 | 0.249 |
| **Therapy，n (%)** |  |  |  |  |
| ERAs | 2 (33.3) | 2(66.7) | 0(0) |  |
| PDE-5is | 1 (16.7) | 0(7.1) | 1(33.3) |  |
| Prostacyclin analogs | 0 (0) | 0(0) | 0(0) |  |
| Combined therapies | 3 (50.0) | 1(33.3) | 2(66.7) |  |
| 6MWD, 6-minute walk distance; BMI, body mass index; CI, cardiac index; ERA, Endothelin receptor antagonist; mPAP, mean pulmonary arterial pressure; mPAWP, mean pulmonary arterial wedge pressure; mRAP, mean right atrial pressure; PDE-5is, phosphodiesterase type 5 inhibitors; PVR, pulmonary vascular resistance; WHO-FC, World Health Organization Functional Classification. **P* <0.05, male patients with IPAH versus female patients with IPAH. | | | | |
